# Influence of Implant Type on Clinical and Radiologic Outcomes: A Cross‐Sectional Private Practice‐Based Analysis

**DOI:** 10.1002/cre2.70411

**Published:** 2026-07-16

**Authors:** Cristian Scognamiglio, Gülce Çakmak, Martin Schimmel, Alessandro Perucchi, Samir Abou‐Ayash

**Affiliations:** ^1^ Private Practice Mendrisio Switzerland; ^2^ Department of Reconstructive Dentistry and Gerodontology, School of Dental Medicine University of Bern Bern Switzerland; ^3^ Department of Prosthodontics Geriatric Dentistry and Craniomandibular Disorders, Charité Universitätsmedizin Berlin, Corporate Member of Freie Universität Berlin and Humboldt‐Universität zu Berlin Berlin Germany; ^4^ Department of Prosthodontics, Faculty of Dentistry Biruni University Istanbul Turkey; ^5^ Department of Prosthodontics and Biomaterials University Medical Center of the Johannes Gutenberg University Mainz Mainz Germany

**Keywords:** BLX, bone level, bone peak, marginal bone alterations, tissue level

## Abstract

**Objectives:**

To compare tissue‐level (TL) and bone‐level X (BLX) implants regarding peri‐implant bone level alterations, survival, and complication rates over a 3‐years.

**Materials and Methods:**

In this retrospective, non‐interventional clinical study, patients that had received either TL or BLX implants (both: Straumann AG), with at least one clinical follow‐up (minimum 6 months), were included. Bone‐level alterations and bone peaks were evaluated at baseline (implant placement) and at follow‐ups from dental radiographs. In addition, clinical follow‐ups included the recording of the presence of keratinized mucosa and the registration of technical complications (e.g., chipping fractures). For statistics, descriptive analyses, *t*‐tests, and a random‐effects linear regression analysis were applied.

**Results:**

A total of 146 implants (73 TL and 73 BLX) were included. TL implants showed a survival rate of 100%, whereas BLX implants showed a survival rate of 98% after 3 years. TL implants showed significant mesial bone gain (0.08 mm, *p* = 0.018) between 2‐ and 3‐year follow‐ups, with a notable difference (0.21 mm, *p* = 0.041) compared to BLX implants. At distal bone levels, TL implants showed initial bone loss (0.12 mm, *p* = 0.020) at 6 months but gained bone (0.07 mm, *p* < 0.001) between 2 and 3 years, whereas BLX implants showed significant distal bone loss (0.15 mm, *p* = 0.038) over 3 years. The number of complications was higher in BLX implants. BLX implants with a band of keratinized mucosa > 2 mm showed smaller bone level alterations.

**Conclusions:**

Both TL and BLX implants performed similarly in terms of clinical and radiological outcomes. However, at the mesial aspect, bone level alterations in TL implants were smaller than in BLX implants, and complications were less frequent. Keratinized mucosa seems especially beneficial in BLX implants. As implant selection was based on clinical indication, the two groups differed in baseline characteristics, and this should be considered when interpreting the between‐group comparisons.

**Abbreviations**
BLX: bone‐level X implant
C/I: crown/implant ratio
FDP: fixed dental prosthesis
IAC: implant‐abutment connection
KM: keratinized mucosa
LoA: limit of agreement
Mo: month
OT/I: width/implant diameter ratio
RB: regular base
RN: regular neck
TL: tissue‐level implant
WB: wide base
WN: wide neck
Y: year

## 1 Introduction

The success of implant rehabilitation depends on achieving functional outcomes while ensuring the long‐term stability and esthetic integrity of peri‐implant soft and hard tissues (Weisgold et al. 1997; Evans and Chen 2008; Albrektsson et al. 1986; Galindo‐Moreno et al. 2022; Kim et al. 2018; Kang et al. 2018). Implant success is influenced by a variety of factors, including anatomical variables, surgical techniques, implant type and design, loading protocols, and the type of prosthetic connections used (Weisgold et al. 1997; Evans and Chen 2008; Albrektsson et al. 1986; Galindo‐Moreno et al. 2022; Kim et al. 2018; Kang et al. 2018).

In terms of anatomical variables, soft tissue biotype, keratinized mucosa (KM) thickness, and buccal bone thickness are decisive (De Rouck et al. 2009). Thin tissue biotype is associated with insufficient blood perfusion (De Rouck et al. 2009) and greater recession (Evans and Chen 2008) and the lack of an adequate KM band (≤ 2 mm) is associated with increased plaque accumulation, tissue inflammation, and mucosal recession (Roos‐Jansåker et al. 2006; Lin et al. 2013; Brito et al. 2014). Bone remodeling after implant placement plays a vital role in maintaining the position and stability of the overlying soft tissues, and a minimum of 1–2 mm of buccal bone thickness is needed to ensure stability and esthetics (Belser et al. 2008; Grunder et al. 2005). The preservation of the bone peaks can contribute greater stability of the soft tissues over time (Kang et al. 2018). Previous studies have reported that implant design (surface, collar, and platform switching) can affect primary stability, bone remodeling, marginal bone loss, soft tissue support, and peri‐implantitis risk (Vela‐Nebot et al. 2006). Therefore, implant selection plays a critical role in achieving good long‐term outcomes. In terms of implant selection, tissue‐level (TL) and bone‐level (BL) implants are two extensively used implant designs (Canullo et al. 2022). TL implants have a prosthetic interface above the alveolar crest, which allows for improved soft tissue adaptation and less microbial contamination via the implant‐abutment connection (Canullo et al. 2015; Tallarico et al. 2018). Previous studies have reported high success and survival rates (98.23% over 10 years) with TL implants (Kim et al. 2018; Kang et al. 2018). Traditionally, TL implants have been used mostly in edentulous posterior areas because of the risks of gingival recession, displaying metal implant collar, and esthetic complications in the anterior areas (Canullo et al. 2022; Menini et al. 2022). However, there is also some retrospective studies that have been showed the use of TL implants as an appropriate option in anterior areas with less crestal bone resorption (Canullo et al. 2022; Hänggi et al. 2005). On the other hand, BL implants have the prosthetic interface at or below the alveolar crest. Therefore, by positioning the implant more apically, BL implants have the advantage of allowing the creation of an ideal emergence profile and optimal esthetic outcomes (Grunder et al. 2005; Buser et al. 2004; Grunder 2000). Hence, in recent years, BL implants have been widely preferred in esthetically demanding cases, particularly in anterior regions and in immediate implant placement and loading (Evans and Chen 2008). For the immediate implantation, good primary stability at the implant placement is critical to prevent micro‐motion and related issues in osseointegration and implant failure (Morton et al. 2023; Hamilton et al. 2023; Greenstein and Cavallaro 2017). Parallel with the increasing numbers of immediate implantations and patient demands in esthetics and immediate function, new bone‐level implant designs or implant surfaces with different topography and chemistry have been introduced (Pellegrini et al. 2018; Rupp et al. 2018). One of these is a fully tapered, self‐cutting implant design with protruding thread geometry and reduced diameter implant neck (Bone level X, BLX; Straumann AG, Basel, Switzerland), which has been introduced to achieve high primary stability and reduce treatment time (Francisco et al. 2021; Parvini et al. 2023; Fiore et al. 2024; Carosi et al. 2023). BLX implants have been especially indicated for immediate implant placement procedures or when there is limited bone quantity (Francisco et al. 2021; Parvini et al. 2023; Fiore et al. 2024; Carosi et al. 2023). A recent cross‐sectional study concluded that the use of BLX implant systems in immediate implant placement and restoration resulted in good peri‐implant health and good pink esthetic outcomes during with a minimum 12‐month follow‐up (Parvini et al. 2023). However, yet there is limited knowledge on the comparison of the clinical and radiological outcomes of TL and BLX implants when used with different implant‐supported prostheses. Therefore, the purpose of this study was to analyze bone remodeling and the implant complications comparing TL and BLX implants when used for different prostheses (single crown, screwed bridge, overdenture). Furthermore, clinical outcomes (survival and success) and potential factors influencing peri‐implant bone level alterations were analyzed. The first null hypothesis was that there would be no difference in terms of peri‐implant bone level alterations around the two implant types at the 3‐year follow‐up. The second null hypothesis was that implant location, type of rehabilitation, the width of keratinized mucosa, would not affect peri‐implant bone level alterations around the two implant types at the 3‐year follow‐up.

## 2 Materials and Methods

### 2.1 Study Design

The present study was a retrospective, non‐interventional, clinical study. The study was approved by the Cantonal Ethics Committee of the Canton of Ticino (reference number: 2024‐00347, Rif CE TI 4545). The study complies with the ethical standards of the Declaration of Helsinki as well as the national legal and regulatory requirements. Patient data (including radiologic data) were extracted from the digital patient records. The participants did not undergo any additional clinical appointments. All participants gave written informed consent before data extraction from their patient records.

### 2.2 Selection and Cataloging of the Study Sample

The first step of the cataloging was to select the patients that had received either TL or BLX implants (both: Straumann AG) with clinical follow‐ups at least 6 months, including radiograph recording. All selected patients had a 6‐month periodic recall for professional dental hygiene. All implants selected for the study had cleanable restorations for the patients to be maintainable.

**Figure 1:**
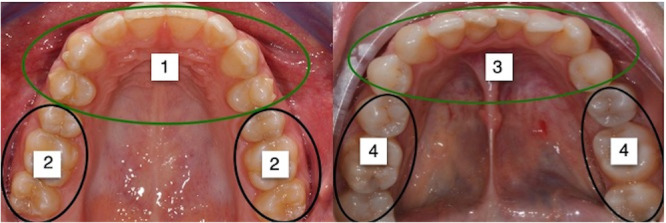
Different implant placement areas used for categorization of the implants. The arch was divided into four regions: anterior superior (teeth 14–24), posterior superior (teeth 15–18 and 25–28), anterior inferior (teeth 34–44), and posterior inferior (teeth 35–38 and 45–48).

The implants were categorized based on the following criteria:

1. Implant placement areas (Figure 1):
  a. Anterior superior (teeth 14–24).
  b. Posterior superior (teeth 15–18 and teeth 25–28).
  c. Anterior inferior (teeth 34–44).
  d. Posterior inferior (teeth 35–38 and teeth 45–48).
2. Date of implant positioning
3. Implant measurements (length and diameter):
  a. BLX Implant: RB (regular base ø3.50, 3.75, 4.00, 4.50 mm) or WB (wide base ø5.00, 5.50, 6.50 mm).
  b. Tissue Level Implant: RN (regular neck ø3.30, 4.10 mm) or WN (wide neck ø4.80 mm).
4. Implant insertion torque [N]
5. Bone augmentation: yes or no
6. Immediate implant placement after extraction: yes or no
7. Immediate loading: yes or no
8. Type of rehabilitation:

1 = Single crown

2 = Fixed dental prosthesis (FDP)

3 = Overdenture

**Figure 2:**
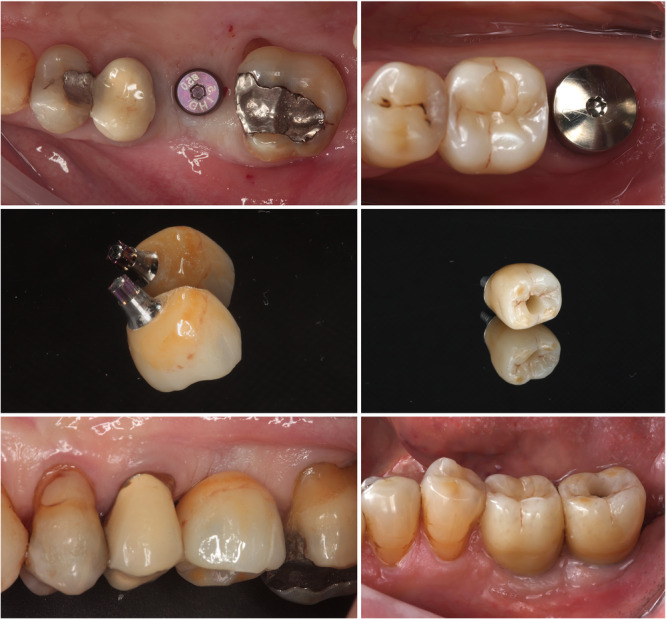
Representative clinical cases of a bone‐level X (BLX) implant and a tissue‐level (TL) implant, respectively.

Figure 2 shows a representative case for BLX and TL implants, respectively.

### 2.3 Surgical and Prosthetic Protocol

All surgical and prosthetic procedures were performed in a single private practice by two experienced clinicians, each with more than 10 years of clinical experience in implant surgery and implant‐supported prosthodontics. For every patient, the same clinician who placed the implant also performed the corresponding prosthetic rehabilitation. Both operators worked according to a common, standardized clinical protocol for surgery, loading, and prosthetic management, and applied identical implant‐positioning criteria for both implant types, in order to minimize operator‐related variability.

All implants were placed using a full‐thickness mucoperiosteal flap and a freehand approach based on preoperative cone‐beam computed tomography (CBCT) planning. Implant three‐dimensional positioning followed a prosthetically driven implant planning and a bio‐restorative concept, (Pedrinaci et al. 2024; Lanis et al. 2025) respecting the recommended minimum distances between adjacent implants and between implants and natural teeth. When implants were placed in healed sites, surgery was performed according to the conventional staged protocol. When tooth extraction was required, the teeth were extracted atraumatically to preserve the extraction site walls; after debridement and rinsing, a collagen cone (Collacone, Botiss Biomaterials, Zossen, Germany) was applied and the site was sutured, and implant placement was performed approximately 3 months after extraction. Guided bone regeneration, using a resorbable collagen membrane (Jason membrane, Botiss Biomaterials, Zossen, Germany) in combination with a xenograft bone substitute, was performed only when a bony defect or insufficient bone volume was present at the time of placement. Immediate loading was carried out only when primary stability of at least 35 Ncm insertion torque was achieved; when the criteria for immediate loading were not met, implants were loaded after a healing period of approximately 12 weeks. The standardized postoperative protocol consisted of chlorhexidine‐based rinses (Perio Plus, Curaden Germany GmbH, Stutensee, Germany) three times daily for 2 weeks, the prescription of analgesics, and suture removal 14 days after surgery. All patients were enrolled in a 6‐month periodic maintenance recall for professional oral hygiene.

### 2.4 Data Collection

All patient data were collected based on the standard follow‐up protocol of the private practice. Intraoral radiographs for analyzing the bone levels/bone peaks mesial and distal to the implants during the routine follow‐ups were recorded using a paralleling technique to obtain reproducible data. In addition, the values of the KM were recorded using a graduated periodontal probe and measuring the band of KM buccal to the implant.

The following variables were recorded. In addition, periimplantitis, mucositis, implant fracture, restoration chipping, and screw loosening presence or absence were recorded.

#### 2.4.1 Bone Level Measurement

The peri‐implant bone levels were analyzed using the software Sidexis (Version 4.2, Dentsply Sirona), offering the possibility to measure distances and angles. For calibration of the measurements, the known inter‐thread distances were used on each radiograph. Every measure of the bone level mesial/distal and bone peak mesial/distal was recorded in the digital chart based on the first measurement (M1).

Analyzing the reproducibility of the radiological measurements, 20 implants of each group were randomly selected, and the radiological measurements were done a second time (M2). Analyzing bone level alterations, the values from baseline were subtracted from the follow‐up visits’ values.

##### 2.4.1.1 BLX Implant Bone Level Measurement

**Figure 3:**
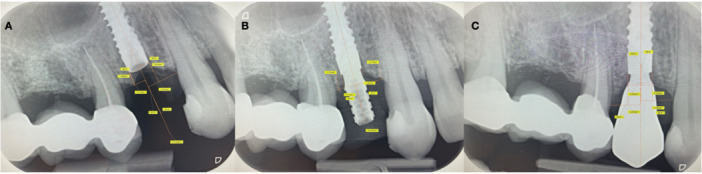
Radiographic bone level measurement for BLX implants. A line was drawn along the central axis of the implant, and a perpendicular reference line was drawn touching the coronal edge of the implant at the mesial and distal points; the vertical distances from this reference line to the bone and to the mesial and distal bone peaks were then measured. (A) Measurement of the implant without a cap screw. (B) Measurement of the implant with a temporary abutment. (C) Measurement of the implant with the final crown.

A line was drawn in the central axis of the implants. Afterward, a perpendicular line from the main central axis of the implant, touching the coronal edge of the implant in the mesial and distal points, was created. Starting from this horizontal line, the vertical distance from the mesial and distal points of the coronal edge of the implant to the bone and the vertical distance from the mesial and distal bone peak to this horizontal reference line were measured (Figure 3A–C).

##### 2.4.1.2 Tissue Level Implant Bone Level Measurement

**Figure 4:**
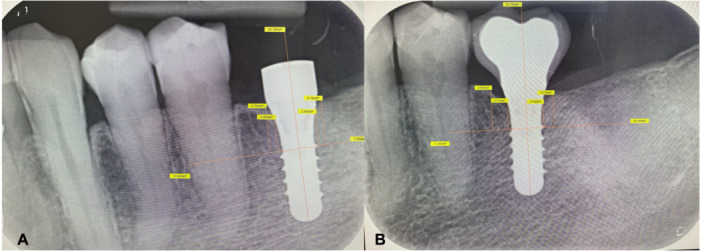
Radiographic bone level measurement for TL implants. A line was drawn along the central axis of the implant, and a perpendicular reference line was drawn touching the most coronal implant thread; the vertical distances from this reference line to the bone and to the mesial and distal bone peaks were then measured. (A) Measurement of the implant with the healing cap. (B) Measurement of the implant with the final crown.

A line was drawn in the central axis of the implants. Afterward, a perpendicular line from the main central axis of the implant was drawn, touching the most coronal implant thread. Starting from this horizontal line, the vertical distance from the mesial and distal points of the implant thread to the bone and the vertical distance from the mesial and distal bone peak to this horizontal reference line were measured (Figure 4A,B).

##### 2.4.1.3 Other Parameters

Peri‐implant parameters were recorded at every follow‐up appointment, including bleeding on probing, probing depths, and the presence of KM, using a periodontal probe. Based on these measurements and the radiological analyses, the implants were categorized for the following failures: periimplantitis (a plaque‐associated pathological condition occurring in tissue around dental implants, characterized by inflammation in the peri‐implant mucosa and subsequent progressive loss of supporting bone (Berglundh et al. 2018)), mucositis (inflammation of the peri‐implant mucosa; the main clinical characteristic of peri‐implant mucositis is bleeding on gentle probing (Berglundh et al. 2018)), fracture, restoration chipping, or screw loosening.

##### 2.4.1.4 Implant Survival and Success Criteria

Implant survival was defined as an implant that remained in situ and functional at the last follow‐up, regardless of biological or technical complications. The survival rate was calculated as the number of surviving implants divided by the total number of implants placed within each group at the corresponding follow‐up. Implant success was defined more strictly, in accordance with commonly applied criteria, (Berglundh et al. 2018) as a surviving implant that additionally showed stable peri‐implant hard and soft tissues, the absence of peri‐implant mucositis or peri‐implantitis (no clinical signs of inflammation, no bleeding on probing, suppuration, increasing probing depths, recession of the mucosal margin, or progressive radiographic bone loss), and the absence of any biological or technical complication during the observation period.

Peri‐implantitis was recorded when an implant presented peri‐implant inflammation with bleeding on probing and/or suppuration, increased probing depth and/or recession of the mucosal margin relative to baseline, in addition to progressive radiographic crestal bone loss exceeding the expected initial remodeling.

### 2.5 Statistical Analysis

Descriptive analyses (mean values, standard deviations, median values, minimum and maximum values) were calculated, describing the peri‐implant bone level data at the different timepoints as well as the differences between the timepoints. The *t*‐test for dependent (intragroup analysis) or independent (intergroup analysis) data was used to test the hypothesis of no difference between the different timepoints within and between the two implant types. For the comparison of the bone level alterations between the two implant types, the *t*‐test was adjusted for the baseline of each implant's bone level values. A random‐effects linear regression analysis was used to test the potential effect of the influencing factors on the peri‐implant bone level alterations. Furthermore, to evaluate intra‐rater reliability for the performed measurements, Bland‐Altman statistics, including limit of agreement (LoA) analyses, were used. All statistical analyses were performed using Stata/IC software (Version 14.2 for Windows) with the alpha level set to 0.05. Because of the retrospective design, no a priori sample size or power calculation was performed; instead, a consecutive sample of all eligible implants with complete baseline and follow‐up records was analyzed. While the sample was adequate to detect statistically significant differences in the primary radiographic outcomes, the secondary multivariable analyses are exploratory in nature, and their findings should be interpreted as hypothesis‐generating.

## 3 Results

### 3.1 Study Population

For the study, 43 male and 44 female patients with an average age of 50 years were selected. Among the 87 patients selected, 77 were healthy, 4 had controlled diabetes, and 6 had high blood pressure, 7 patients were smokers and none of them had a history of periodontal disease. The total number of implants selected for the study was 146, divided into 73 TL (Straumann AG, Basel, Switzerland) and 73 BLX (Straumann AG, Basel, Switzerland) implants. Table 1 shows patient and implant related data.

**Table 1:** Patient and implant related data.

		Tissue level (TL)	Bone level (BLX)
Patient factors	Males (*n* = 43)	73: baseline, 1 year	73: baseline, 1 year
	Females (*n* = 44)	72: 2 years	68: 2 years
		69: 3 years	48: 3 years
Recipient‐site factors	Anterior superior (1)	1: 2	1: 19
	Posterior superior (2)	2: 13	2: 44
	Anterior inferior (3)	3: 8	3: 3
	Posterior inferior (4)	4: 50	4: 7
GBR	Yes	17	56
	No	56	17
Implant diameter	3.3 mm	19	N/A
	3.5 mm	N/A	8
	3.75 mm	N/A	22
	4 mm	N/A	7
	4.1 mm	38	N/A
	4.5 mm	N/A	26
	4.8 mm	16	N/A
	5 mm	N/A	3
	5.5 mm	N/A	6
	6.5 mm	N/A	1
Implant length	6 mm	N/A	2
	8 mm	10	18
	10 mm	42	28
	12 mm	21	22
	14 mm	N/A	3
Insertion torque (Ncm)	< 34	43	N/A
	35–39	28	1
	40–49	1	18
	≥ 50	1	54
Immediate placement	Yes	0	24
	No	73	49
Immediate loading	Yes	0	27
	No	73	46
Restoration type	Single crown	55	40
	Fixed dental prosthesis (FDP)	10	22
	Overdenture	8	11
Keratinized mucosa	< 2 mm	0	10
	≥ 2 mm	73	63
Implant failure	Yes	0	2 in 2 years follow up
			1 in 3 years follow up
	No	73	73
Periimplantitis	Yes	0	0
	No	73	73
Mucositis	Yes	0	1 in 3 years follow up
	No	73	73
Implant fracture	Yes	0	0
	No	73	73
Chipping	Yes	0	1 in 2 years follow up
			2 in 3 years follow up
	No	73	70
Screw loosening	Yes	0	0
	No	73	73

### 3.2 Reproducibility and Reliability of Bone Level Alteration Analyses

Table 2 shows the reliability of the obtained data, considering the different measurements (mesial bone/distal bone/peak mesial/peak distal) in BLX and TL implants.

**Table 2:** Overview of the reproducibility analysis.

			*n*	Mw ± SD	Median	Min–Max	*p* value
Mesial	BLX	M1	20	2.10 ± 0.86	2.02	0.26–3.56	
		M2	20	2.10 ± 0.86	2.04	0.28–3.58	
		M1–M2	20	0.00 ± 0.02	−0.01	−0.04 to 0.03	0.302
	TL	M1	21	2.77 ± 0.82	3.04	0.45–3.89	
		M2	21	2.77 ± 0.82	3.07	0.47–3.88	
		M1–M2	21	0.00 ± 0.03	−0.01	−0.04 to 0.06	0.821
Distal	BLX	M1	20	2.11 ± 1.03	2.08	0.30–3.78	
		M2	20	2.11 ± 1.03	2.07	0.33–3.78	
		M1–M2	20	0.00 ± 0.02	0.00	−0.03 to 0.04	1.000
	TL	M1	21	2.78 ± 0.79	2.93	0.45–3.78	
		M2	21	2.78 ± 0.79	2.92	0.47–3.78	
		M1–M2	21	0.00 ± 0.02	0.00	−0.04 to 0.04	0.505
Peak mesial	BLX	M1	20	3.38 ± 1.47	3.29	0.30–7.15	
		M2	20	3.39 ± 1.47	3.30	0.33–7.18	
		M1–M2	20	−0.01 ± 0.02	−0.01	–0.05 to 0.02	0.028
	TL	M1	21	4.03 ± 1.26	3.86	0.89–5.50	
		M2	21	4.05 ± 1.27	3.85	0.85–5.54	
		M1–M2	21	−0.01 ± 0.02	−0.02	−0.04 to 0.04	0.001
Peak distal	BLX	M1	20	3.00 ± 1.47	2.87	0.33–5.68	
		M2	20	3.01 ± 1.47	2.87	0.35–5.69	
		M1–M2	20	−0.00 ± 0.02	−0.01	−0.03 to 0.03	0.225
	TL	M1	21	3.54 ± 0.99	3.59	0.94–4.97	
		M2	21	3.55 ± 0.98	3.56	0.98–4.99	
		M1–M2	21	−0.01 ± 0.03	−0.02	−0.06 to 0.04	0.011

Abbreviations: M1, first measurement; M2, controlling measurement; M1–M2, difference between M1 and M2; Mw, mean value; SD, standard deviation.

In terms of mesial and distal bone level alterations, there were no significant differences in terms of the reliability of the obtained data in both implant types (*p* ≥ 0.0.302). In terms of mesial bone peak, a statistically significant difference was found in both implants, while in terms of distal bone peak, a statistically significant difference was only found in TL implants with a mean difference between measurement (M1) and measurement 2 (M2) of 0.01 + /− 0.2 mm (*p* ≤ 0.028). The maximum limit of agreement considering all measurements was 0.07 mm (Table 3).

**Table 3:** Limits of agreement.

		Mw ± SD	95%‐CI	LoA
Mesial	BLX	−0.00 ± 0.02	−0.02; 0.01	−0.05; 0.04
	TL	−0.00 ± 0.03	−0.01; 0.01	−0.06; 0.05
Distal	BLX	0.00 ± 0.02	−0.01; 0.01	−0.04; 0.04
	TL	−0.00 ± 0.02	−0.01; 0.01	−0.05; 0.04
Peak mesial	BLX	−0.01 ± 0.02	−0.02; −0.00	−0.05; 0.03
	TL	−0.01 ± 0.02	−0.02; −0.00	−0.05; 0.02
Peak distal	BLX	−0.00 ± 0.02	−0.01; 0.00	−0.04; 0.03
	TL	−0.01 ± 0.03	−0.03; −0.00	−0.07; 0.04

Abbreviations: 95%‐CI, 95% confidence interval; LoA, limit of agreement; Mw, mean value; SD, standard deviation.

### 3.3 Comparison of BLX and TL Implants

#### 3.3.1 Mesial and Distal Bone Level and Bone Peak Alterations

In terms of mesial bone level alterations (Table 4), TL implants had a significant bone increase (0.08 mm, *p* = 0.018) between 2‐year and 3‐year follow‐ups. After 3 years, bone loss was significantly higher in BLX implants compared to TL implants (*p* = 0.041). In terms of distal bone level alterations, TL implants showed a significant bone loss after 6 months, while it had a significant bone increase between 2‐year and 3‐year follow‐ups (*p* < 0.001). BLX implants showed a significant distal bone loss (0.15 mm) at the 3‐year follow‐up. Between 1‐year and 2‐year follow‐ups the bone level alterations were significantly higher in BLX implants (*p* = 0.043), however, no significant difference after 3 years was found (*p* = 0.100). In terms of mesial and distal bone peak level alterations (Table 5), there were no significant difference in terms of mesial or distal bone peak level alterations between BLX and TL implants in any time period (*p* ≥ 0.065).

**Table 4:** Mesial and distal bone level alterations in BLX and TL implants considering different follow‐up time‐points.

			*n*	Mw ± SD	Median	Min–Max	Comparison TL vs. BLX
Mesial	BL‐6 Mo	BLX	73	−0.08 ± 0.38	0.03	−1.78 to 0.82	*p* = 0.672
		TL	73	−0.05 ± 0.43	0.02	−1.90 to 1.07	0.03 [−0.11; 0.16]
	6 Mo‐1Y	BLX	73	0.00 ± 0.27	0.02	−0.95 to 0.65	*p* = 0.602
		TL	73	0.03 ± 0.26	0.02	−0.98 to 0.73	0.02 [−0.06; 0.11]
	1 Y‐2 Y	BLX	68	−0.01 ± 0.33	0.02	−0.93 to 0.88	*p* = 0.451
		TL	72	0.03 ± 0.23	0.02	−0.62 to 0.65	0.04 [−0.06; 0.13]
	2 Y‐ 3 Y	BLX	48	0.02 ± 0.28	0.01	−0.75 to 0.75	*p* = 0.285
		TL	69	0.08^A^ ± 0.28	0.03	−0.71 to 1.27	0.06 [−0.05; 0.16]
	BL‐3 Y	BLX	48	−0.13 ± 0.52	0.02	−1.68 to 1.08	*p* = 0.041
		TL	69	0.08 ± 0.57	0.08	−1.68 to 1.91	0.21 [0.01; 0.41]
Distal	BL‐6 Mo	BLX	73	−0.03 ± 0.41	0.02	−2.26 to 1.15	*p* = 0.203
		TL	73	−0.12^B^ ± 0.42	0.01	−1.94 to 1.14	−0.09 [−0.22; 0.05]
	6 Mo‐1Y	BLX	73	−0.05 ± 0.37	0.02	−1.53 to 0.75	*p* = 0.190
		TL	73	0.02 ± 0.29	0.02	−0.79 to 1.14	0.07 [−0.04; 0.18]
	1 Y‐2 Y	BLX	68	−0.07 ± 0.34	0.01	−0.96 to 0.70	*p* = 0.043
		TL	72	0.04 ± 0.28	0.03	−1.08 to 0.73	0.11 [0.00; 0.21]
	2 Y‐ 3 Y	BLX	48	0.08 ± 0.40	0.02	−0.89 to 1.85	*p* = 0.846
		TL	69	0.07^C^ ± 0.16	0.03	−0.08 to 0.85	−0.01 [−0.13; 0.11]
	BL‐3 Y	BLX	48	−0.15^D^ ± 0.47	−0.07	−1.54 to 1.00	*p* = 0.100
		TL	69	−0.01 ± 0.41	0.09	−1.12 to 0.86	0.14 [−0.03; 0.31]

*Note:* A positive number in column Mw indicates bone growth, while a negative value indicates bone loss. A: *p* = 0.018, B: *p* = 0.020, C: *p* < 0.001, D: *p* = 0.038.

Abbreviations: Mw, mean value; N, number of implants; SD, standard deviation.

**Table 5:** Mesial and distal bone peak level alterations in BLX and TL implants considering different follow‐up time‐points.

			*n*	Mw ± SD	Median	Min–Max	Comparison TL vs. BLX
Mesial	BL‐6 Mo	BLX	73	−0.11 ± 0.54	0.02	−2.38 to 0.86	*p* = 0.311
		TL	73	−0.20^A^ ± 0.52	0.00	−2.29 to 1.56	−0.09 [−0.26; 0.08]
	6 Mo‐1 Y	BLX	73	0.02 ± 0.42	0.04	−1.51 to 1.29	*p* = 0.901
		TL	73	0.03 ± 0.35	0.02	−0.85 to 1.20	0.01 [−0.12; 0.13]
	1 Y‐2 Y	BLX	68	0.03 ± 0.43	0.05	−1.29 to 1.20	*p* = 0.817
		TL	72	0.05 ± 0.26	0.03	−0.61 to 1.11	0.01 [−0.10; 0.13]
	2 Y‐3 Y	BLX	48	0.08 ± 0.34	0.02	−0.54 to 1.19	*p* = 0.285
		TL	69	0.02 ± 0.28	0.03	−1.17 to 1.09	−0.06 [−0.18; 0.05]
	BL‐3 Y	BLX	48	−0.06 ± 0.77	0.13	−3.03 to 1.44	*p* = 0.723
		TL	69	−0.11 ± 0.61	0.05	−2.29 to 1.29	−0.05 [−0.31; 0.22]
Distal	BL‐6 Mo	BLX	73	−0.05 ± 0.59	0.02	−2.60 to 2.10	*p* = 0.065
		TL	73	−0.21^B^ ± 0.47	−0.05	−1.78 to 0.68	−0.16 [−0.34; 0.01]
	6 Mo‐1 Y	BLX	73	−0.07 ± 0.57	0.02	−2.07 to 2.29	*p* = 0.221
		TL	73	0.03 ± 0.35	0.02	−1.24 to 1.29	0.10 [−0.06; 0.25]
	1 Y‐2 Y	BLX	68	−0.02 ± 0.48	0.02	−1.40 to 2.20	*p* = 0.700
		TL	72	0.01 ± 0.32	0.03	−1.44 to 1.09	0.03 [−0.11; 0.16]
	2 Y‐3 Y	BLX	48	0.04 ± 0.41	0.03	−1.53 to 1.41	*p* = 0.529
		TL	69	0.08^C^ ± 0.20	0.03	−0.10 to 1.24	0.04 [−0.09; 0.17]
	BL‐3 Y	BLX	48	−0.10 ± 0.65	0.00	−2.00 to 1.34	*p* = 0.864
		TL	69	−0.12 ± 0.51	0.06	−1.85 to 1.24	−0.02 [−0.24; 0.20]

*Note:* A, *p* = 0.002, B, *p* < 0.001, C, *p* = 0.002.

Abbreviations: Mw, mean value; N, number of implants; SD, standard deviation.

Table 6 gives an overview of all potential influencing factors. In terms of potential influencing factors for marginal bone level alterations, a significant effect of the implant location was observed in TL implants but not in BLX implants. In TL implants, the marginal bone level alterations were significantly higher in maxillary anterior sites compared to all other locations (*p* ≤ 0.001) at the mesial implant aspect and in the maxillary anterior sites compared to the mandibular posterior sites at the distal aspect (*p* = 0.028). Marginal bone level alterations were more pronounced in implants supporting overdentures compared to single crowns and FDPs, independent of the implant type (*p* ≤ 0.043). BLX implants supporting FDPs had higher marginal bone level alterations at the mesial aspects compared to implants supporting single crowns (*p* = 0.010). TL implants supporting FDPs had smaller mesial and distal bone level alterations compared to single crowns (*p* ≤ 0.022). BLX implants with a band of keratinized mucosa > 2 mm showed smaller bone level alterations in the mesial aspect compared to implants with less keratinized mucosa (*p* = 0.041). Since TL implants had a band of keratinized mucosa of > 2 mm, no analysis for TL implants could be executed.

**Table 6:** Potential influencing factors for marginal bone level alterations. Position: 1. Anterior superior (from tooth 14 to tooth 24), 2. Posterior superior (from tooth 15 to tooth 18 and from tooth 25 to tooth 28), 3. Anterior inferior (from tooth 34 to tooth 44), 4. Posterior inferior (from tooth 35 to tooth 38 and from tooth 45 to tooth 48). Dimension: 0: RB (regular base ø3,50/3,75/4,00/4,50); 1: WB (wide base ø5,00/5,50/6,50). Bone augmentation, immediate implant placement, immediate loading: 1: yes, 0: no, type of rehabilitation: 1 = Single Crown, 2 = Fixed Dental Prosthesis (FDP), 3 = Overdenture. Positive values indicate higher bone level alterations in the first group.

		BLX mesial (*n* = 335)	TL mesial (*n* = 360)	BLX distal (*n* = 335)	TL distal (*n* = 360)
Position	2 vs. 1	0.13 [−0.39; 0.65] *p* = 0.618	−0.99 [−1.55; −0.43] *p* = 0.001	−0.08 [−0.59; 0.42] *p* = 0.752	−0.40 [−0.84; 0.04] *p* = 0.075
	3 vs. 1	−0.45 [−1.71; 0.80] *p* = 0.482	−1.22 [−1.66; −0.78] *p* < 0.001	−0.26 [−1.48; 0.96] *p* = 0.671	−0.27 [−0.64; 0.10] *p* = 0.146
	4 vs. 1	−0.64 [−1.38; 0.10] *p* = 0.091	−1.22 [−1.80; −0.63] *p* < 0.001	−0.08 [−0.73; 0.57] *p* = 0.813	−0.52 [−0.99; −0.06] *p* = 0.028
Dimension	1 vs. 0	0.05 [−0.38; 0.49] *p* = 0.807	0.15 [−0.05; 0.35] *p* = 0.143	0.06 [−0.38; 0.49] *p* = 0.801	0.21 [−0.00; 0.41] *p* = 0.054
Torque	<= 50 (< = 30)	0.37 [−0.04; 0.78] *p* = 0.080	−0.06 [−0.37; 0.24] *p* = 0.695	0.21 [−0.21; 0.62] *p* = 0.328	0.09 [−0.18; 0.37] *p* = 0.500
Augmentation	1 vs. 0	−0.17 [−0.58; 0.24] *p* = 0.422	0.08 [−0.26; 0.42] *p* = 0.637	−0.15 [−0.53; 0.23] *p* = 0.432	0.01 [−0.25; 0.27] *p* = 0.932
Immediate placement	1 vs. 0	−0.05 [−0.54; 0.43] *p* = 0.823	—	0.42 [−0.07; 0.92] *p* = 0.093	—
Immediate loading	1 vs. 0	−0.35 [−0.78; 0.07] *p* = 0.105	—	−0.26 [−0.73; 0.21] *p* = 0.275	—
Type of rehabilitation	2 vs. 1	0.67 [0.16; 1.18] *p* = 0.010	−0.43 [−0.81; −0.06] *p* = 0.022	0.42 [−0.17; 1.01] *p* = 0.164	−0.45 [−0.72; −0.19] *p* = 0.001
	3 vs. 1	1.02 [0.39; 1.64] *p* = 0.001	0.71 [0.02; 1.40] *p* = 0.043	1.08 [0.52; 1.64] *p* < 0.001	0.48 [−0.20; 1.16] *p* = 0.171
	3 vs. 2	0.34 [−0.14; 0.82] *p* = 0.160	1.14 [0.51; 1.78] *p* < 0.001	0.66 [0.16; 1.16] *p* = 0.010	0.93 [0.31; 1.54] *p* = 0.003
Mucosa	<= 2 vs. > 2	0.82 [0.03; 1.61] *p* = 0.041	—	0.65 [−0.16; 1.46] *p* = 0.114	—

### 3.4 Complications

Table 7 summarizes the complications over the different time‐periods. At the 3‐year follow‐up, the total number of complications was significantly higher in BLX implants compared to TL implants (8.2% vs. 0.0% *p* = 0.028). Implant failures were only found in BLX implants (1 implant), however, without a statistically significant difference compared to TL implants (*p* = 0.42).

**Table 7:** Complications over the different time‐periods.

		Implant failure	Peri‐implantitis	Mucositis	Implant fracture	Chipping	Screw loose	Any complication
6 Mo	BLX	0/73 (0.0%)	0/73 (0.0%)	0/73 (0.0%)	0/73 (0.0%)	0/73 (0.0%)	0/73 (0.0%)	0/73 (0.0%)
	TL	0/73 (0.0%)	0/73 (0.0%)	0/73 (0.0%)	0/73 (0.0%)	0/73 (0.0%)	0/73 (0.0%)	0/73 (0.0%)
1 Y	BLX	0/73 (0.0%)	0/73 (0.0%)	0/73 (0.0%)	0/73 (0.0%)	0/73 (0.0%)	0/73 (0.0%)	0/73 (0.0%)
	TL	0/73 (0.0%)	0/73 (0.0%)	0/73 (0.0%)	0/73 (0.0%)	0/73 (0.0%)	0/73 (0.0%)	0/73 (0.0%)
2 Y	BLX	2/70 (2.9%)	0/68 (0.0%)	0/68 (0.0%)	0/68 (0.0%)	1/68 (1.5%)	0/68 (0.0%)	3/70 (4.3%)
	TL	0/72 (0.0%) *p* = 0.24	0/72 (0.0%)	0/72 (0.0%)	0/72 (0.0%)	0/72 (0.0%) *p* = 0.49	0/72 (0.0%)	0/72 (0.0%) *p* = 0.117
3 Y	BLX	1/49 (2.0%)	0/48 (0.0%)	1/48 (2.1%)	0/48 (0.0%)	2/48 (4.2%)	0/48 (0.0%)	4/49 (8.2%)
	TL	0/69 (0.0%) *p* = 0.42	0/69 (0.0%)	0/69 (0.0%) *p* = 0.41	0/69 (0.0%)	0/69 (0.0%) *p* = 0.17	0/69 (0.0%)	0/69 (0.0%) *p* = 0.028

## 4 Discussion

The present study analyzed the bone remodeling and the implant complications comparing two different types of implants, TL and BLX, after 3 years of function. TL implants had 100% survival rate after 3 years, while BLX implants had 97% survival rate after 2 years and 98% survival rate after 3 years (increase due to dropouts). At the mesial aspect, TL implants showed less bone level alteration compared to BLX implants after 3 years. Therefore, the first hypothesis of no difference in terms of peri‐implant bone level alterations around the two implants was rejected. The marginal bone level alterations were influenced by the implant location, type of rehabilitation, and the width of keratinized mucosa. In addition, implant type affected complications. Therefore, the second null hypothesis of no influence of these factors was also rejected.

The demonstrated survival rates are consisted with previously published systematic review and meta‐analysis that reported > 97% cumulative survival rates in BL and TL implants in short‐, mid‐ and long‐term follow‐ups (Schoenbaum et al. 2023). High‐long‐term survival rates for TL implants have been described in the literature, (Kim et al. 2018; Schoenbaum et al. 2023) however, no long‐ or mid‐term follow‐up data for BLX were available to compare the present results to. In the present study, the maximum bone level alterations were 0.15 ± 0.47 mm for BLX implants and 0.12 ± 0.42 mm for TL implants, regardless of the time frame, which are within clinically acceptable limits reported by Albrektsson et al. (1986) (except the first year, annual bone loss of 0.2 mm), Derks et al. (2016) (> 0.5 mm bone loss at years 2 and 3), and Galindo‐Galindo‐Moreno et al. (2022) (> 0.5 mm of radiographic marginal bone loss 6 months after loading). The observed marginal bone level alterations in the present study are lower than in a systematic review and meta‐analysis of 45,347 implants reporting mid‐term (2 to 5 years) and long‐term (5 years) bone level alterations as 0.4 mm and 0.43 mm in TL implants and 0.45 mm and 0.44 mm in BL implants with an internal, narrow cone (< 45°) and 0.73 mm and 0.95 mm in BL implants with an internal, wide cone (≥ 45°) designs, respectively (Schoenbaum et al. 2023). This review (Schoenbaum et al. 2023) has reported that implant‐abutment connection (IAC) plays a significant role on alterations in marginal bone levels. When the bone level and bone peak level alterations between BLX and TL implants are compared, the maximum difference was 0.21 mm in the mesial aspect after 3 years, which can be considered within acceptable limits. The reason for the higher bone loss in the BLX group may be attributed to the implant design and IAC type. The IAC in BLX implants is closer to the bone crest, resulting in potential micro leakage or bone irritation by screwing and unscrewing of implant parts‐in and screw‐out, at the bone level (Zipprich et al. 2007). In TL implants, the connection is at the level of the soft tissues and the risk of contamination is at a larger distance from the bone, (Kim et al. 2018; Kang et al. 2018) resulting in smaller bone‐level alterations. However, there were significant initial (after 6 months) bone level and bone peak alterations in TL implants which were not observed in the BLX group. Those alterations could either be attributed to the physiological remodeling that occurs naturally, the pressure that the machined implant neck can produce on the surrounding bone, particularly when placed deeper in the anterior region, or the bone remodeling that occurs when the machined neck is placed subcrestally, which has reduced osteoconductive surface properties (Albrektsson et al. 1986; Kim et al. 2018; Kang et al. 2018; Canullo et al. 2022; Schwarz et al. 2014; Buser et al. 1991; Matar et al. 2023). However, considering the small bone level alterations in the TL group after 3 years, these initial bone level alterations may be considered a temporary phenomenon due to the implant's macro‐design. From a clinical perspective, both implants appear to perform similarly in terms of peri‐implant bone levels and bone peak alterations, given the small difference between the two groups.

The implant location significantly influenced marginal bone level alterations, particularly in TL implants. TL implants exhibited greater marginal bone level alterations in the anterior maxilla compared to other locations. This finding may be related to the fact that TL implants in anterior sites are placed more deeply inside the bone to prevent the implant shoulder from being exposed to the oral cavity, overcoming potential esthetic shortcomings. Kim et al. (2018) reported that the location of the TL implants plays a significant role in the success, but they reported increased failure rates in the mandibular anterior region. Although the implants had similar outcomes in peri‐implant bone stability in the present study, it may be suggested to use TL implants rather in posterior regions, in which subcrestal implant placement to prevent aesthetic can be omitted. The results of the present study, demonstrating smaller bone level alterations with BLX implants in anterior sites are in accordance with previous studies, suggesting that BL implants are generally more preferred for use in the anterior region (Canullo et al. 2022; Menini et al. 2022). For the interpretation of the results, it should be noted that there were statistically significant differences between repeated bone‐level and bone peak measurements of the same implant. Nevertheless, the reliability and the reproducibility can be considered as high. The mean differences between the two measurements were 0.01 mm or 0.02 mm, respectively. Determining such small differences clinically is not possible. Furthermore, the clinical effect of these measurement differences in peri‐implant bone level alterations is questionable. Although, there was a statistically significant difference between the repeated measurements.

Regarding the restoration type, data shows that TL implants perform especially well in screwed FDPs, as well as in overdentures, compared to the BLX implants, while BLX perform well in single crowns. This difference can be attributed to the IAC difference and difference between the design of ti‐base abutments used for crowns and bridges, and differences in restoration design. The IAC in BLX implants is a pure conical connection without vertical seating of the abutments on the implant shoulder. Previous studies have demonstrated high stability in such conical connections, leading to tight sealing between implant and abutment (Fiore et al. 2024; Çakmak et al. 2023). Such connections are especially useful in single unit restorations (Çakmak et al. 2023). However, in multiple unit‐restorations the missing vertical seating on the implant shoulder may lead to vertical displacement in one of the implants leading to a micro‐gap between implant and abutment, or may cause unfavorable tension when the restoration is not seated exactly in the same way, as it was seated for the manufacturing of the restoration (Cascos et al. 2023; Zipprich et al. 2024; Blum et al. 2015). Furthermore, Rohr et al. (2025) reported that the failure modes are different between TL and BL implants and the design of the Ti‐base abutments can affect the distribution of compressive loads and related failure modes and stability of implant‐supported cantilevered prostheses. In a 6‐year retrospective study, Ozgur et al. (2016) reported that excessive crown/implant (C/I) ratio and occlusal table width/implant diameter (OT/I) ratio increased marginal bone loss. A systematic review reported that height abutment can influence early bone loss around BL implants, while its effects on early and late bone loss around TL and late bone loss around BL implants are not clear (Chen et al. 2019). Hence, the difference in restoration morphology and abutment height might have played a role in the marginal bone loss. According to the present study results, TL implants can be especially recommended for multiple‐unit restorations.

The immediate implant placement and immediate loading showed no significant effect on the bone level changes in BLX implants, supporting the suitability of this implant in such cases. This outcome may be attributed to the self‐cutting tapered implant design, which has been reported to provide an unconventional mechanism of bone engagement at the apical and central aspects of the implant through compacting and densifying the peri‐implant bone during insertion (Francisco et al. 2021; Emmert et al. 2021). Supporting present study findings, Parvini et al. (2023) reported that BLX and Ankylos implant systems resulted in comparable peri‐implant health and good pink esthetic outcomes for immediate implant placement and restoration in 52 implants with 12 months of follow up. Carosi et al. (2023) reported that the use of BLX implants in immediate placement and restoration resulted in good primary stability, a high implant survival rate, and favorable radiographic and esthetic outcomes in 37 implants with 12 months of follow‐up. There is debate in the literature in terms of the clinical significance of KM for preserving the stability of the peri‐implant hard and soft tissues (Ramanauskaite et al. 2022; Ravidà et al. 2022). However, in the present study, BLX implants with a keratinized mucosa band wider than 2 mm exhibited less bone level alteration compared to implants with a narrower band of keratinized mucosa.

Beyond the width of the keratinized mucosa, the vertical soft tissue height (mucosal thickness) is a further determinant of crestal bone stability that warrants consideration. Prospective clinical evidence has shown that when the vertical mucosal thickness at the crest is thin (≤ 2 mm), significantly greater early crestal bone loss may occur, even when the implant–abutment interface is positioned supracrestally, whereas a thick mucosa is associated with more limited bone remodeling (Linkevicius et al. 2009; Suárez‐López del Amo et al. 2016; Puisys and Linkevicius 2015). This concept is relevant to the different treatment rationale of the two implant designs evaluated here. In TL implants, the smooth transmucosal collar itself establishes the supracrestal soft tissue component, keeping the prosthetic interface away from the bone crest. In BLX implants, this component must instead be reproduced through three‐dimensional implant positioning; in thin biotypes the implant is therefore placed more apically (subcrestally) to allow for an adequate supracrestal tissue height, which may in turn contribute to the initial bone remodeling that is observed when a machined or polished surface is positioned below the crest. Because the vertical soft tissue height was not systematically recorded in the retrospective records used in the present study, its specific contribution to the observed bone level alterations could not be quantified. Future prospective comparisons of TL and BLX implants should include standardized measurement of the vertical mucosal thickness.

The implant surface treatment and the microgeometry of the implant collar may also influence marginal bone stability and the peri‐implant soft tissue response. Surface topography and chemistry modulate osseointegration and peri‐implant bone remodeling, (Pellegrini et al. 2018; Rupp et al. 2018) while the configuration of the transmucosal collar can affect the nature of the soft tissue attachment. Microtextured or laser‐microgrooved collar and abutment surfaces have been reported to favor a functionally oriented connective tissue attachment that resembles that of the natural dentition and may act as a physiologic barrier against apical migration of the junctional epithelium, thereby contributing to crestal bone preservation (Spinelli et al. 2023; Nevins et al. 2008). The TL and BLX implants compared in the present study differ not only in their implant–abutment connection but also in their collar configuration and machined‐neck characteristics, and these differences may have contributed to the small between‐group differences in marginal bone level alterations observed here. As collar microgeometry and surface characteristics were not experimental variables in this retrospective analysis, their individual contribution could not be isolated and represents a relevant direction for future research.

The results show that the frequency of complications was higher with BLX implants (8.2%) compared to TL implants (0.0%) after a 3‐year follow‐up. For BLX implants, two implant failures and chipping in one case were reported after 2 years, while after 3 years, there was one additional implant failure, one case of mucositis, and two cases of chipping. In contrast, no complications were observed with TL implants. This discrepancy may be attributed to the fact that BLX implants are often used in more demanding clinical cases, particularly in scenarios requiring high esthetic demands. Fiore et al. (2024) reported Ti‐base screw loosening in 4 implants among 73 BLX implants after 4 years of follow‐up. Contrarily, screw loosening was not observed in the present study. The higher frequency of chippings in the BLX group may be related to the tension, that can be created due to the seating difficulties in conical connection IACs, described above.

The present study has certain limitations. Due to the retrospective nature of the study, there was no standardized treatment protocol, defining, for example the implant lengths and diameters, the fabrication of an individual radiological guide, or the restorative procedures. Furthermore, since the study was carried out in a private practice setting, inclusion of patients was not as standardized as in a university setting. However, this can also be considered a strength of the study, as it represents the conditions private practitioners have to face on a daily basis. The high drop‐out rate, especially in the BLX group should also be taken into account. Additionally, the relatively small sample size and the imbalance in implant distribution between anterior and posterior regions may limit the generalizability of the results. Because implant selection was indication‐driven rather than randomized, the two groups were not balanced and differed in their baseline characteristics, with the BLX group treated, on average, under more demanding clinical conditions (more frequent bone augmentation, immediate placement, and immediate loading); the between‐group comparisons should therefore be interpreted as indication‐specific rather than as a direct head‐to‐head superiority of one implant type. In addition, the multivariable regression analyses, which include a larger number of co‐variables, are exploratory and may be underpowered; accordingly, these findings should be regarded as hypothesis‐generating, whereas the primary intra‐ and intergroup radiographic comparisons are supported by the observed statistically significant effects.

## 5 Conclusion

Within the limitations of the present study, both TL and BLX implants demonstrated favorable clinical and radiological outcomes over a 3‐year follow‐up. However, higher complication rates with BLX compared to TL implants should be considered. As implant selection was based on clinical indication, the two groups differed in baseline characteristics, and this should be considered when interpreting the between‐group comparisons. The presence of an adequate band of keratinized mucosa appears beneficial, particularly for BLX implants. Prospective, randomized studies with balanced groups and standardized protocols are needed to confirm these indication‐specific observations.

## Author Contributions

Conceptualization: Cristian Scognamiglio, Samir Abou‐Ayash, Gülce Çakmak, and Alessandro Perucchi. Methodology: Cristian Scognamiglio, Alessandro Perucchi, and Samir Abou‐Ayash. Software: Cristian Scognamiglio, Samir Abou‐Ayash, Gülce Çakmak, and Alessandro Perucchi. Validation: Cristian Scognamiglio, Samir Abou‐Ayash, Alessandro Perucchi, and Martin Schimmel. Formal analysis: Cristian Scognamiglio, Samir Abou‐Ayash, and Alessandro Perucchi. Investigation: Cristian Scognamiglio, Samir Abou‐Ayash, and Alessandro Perucchi. Resources: Cristian Scognamiglio, Samir Abou‐Ayash, Alessandro Perucchi, and Martin Schimmel. Data curation: Cristian Scognamiglio, Samir Abou‐Ayash, Gülce Çakmak, and Alessandro Perucchi. Writing – original draft: Cristian Scognamiglio, Samir Abou‐Ayash, and Gülce Çakmak. Writing – review and editing: Cristian Scognamiglio, Samir Abou‐Ayash, Gülce Çakmak, and Martin Schimmel. Visualization: Samir Abou‐Ayash. Supervision: Samir Abou‐Ayash and Martin Schimmel. Project administration: Cristian Scognamiglio, Samir Abou‐Ayash, Alessandro Perucchi, and Martin Schimmel. All authors have read and approved the final version of the manuscript.

## Funding

The authors have nothing to report.

## Ethics Statement

The study was approved by the Cantonal Ethics Committee of the Canton of Ticino (reference number: 2024‐00347, Rif CE TI 4545). Informed consent was obtained from all participants prior to the extraction of data from their patient records. Patient data, including radiologic records, were retrieved from digital medical records. As this was a retrospective, non‐interventional clinical study, no additional clinical appointments or procedures were required for the participants. All procedures were conducted in accordance with the ethical standards of the institutional research committee and with the 1964 Declaration of Helsinki and its later amendments, as well as national legal and regulatory requirements.

## Conflicts of Interest

The authors declare no conflicts of interest.

## Supporting information


Supporting File

## Data Availability

Data of current study are available from the corresponding author on reasonable request. However, for privacy reasons, no individual data allowing identification of participants (e.g., videos) can be provided.
